# Case of Purpura

**Published:** 1826-11

**Authors:** 

**Affiliations:** St. George's Hospital.


### *Case of Purpura.* Treated by Dr. Chambers, at St. George's Hospital.

The following case illustrates distinctly the inflammatory nature of one variety of Purpura.

March 25, 1826.—Mary Sibbett, aged sixteen, from Chelsea; of fair complexion, and flabby habit of body. Has spots of purpura on the legs, thighs, and fore-arms; and, on the right arm, some appearance of erythema nodosum. Pulse full and frequent; tongue clean; bowels open; urine natural. The catamenia have not yet appeared.

She has been ill nine months with repeated crops of hemorrhagic spots. She says they appear first like elevated pimples, and after a day or two put on their present aspect. She attributes her complaints to her having drunk (just before her first attack) cold water when she was very warm. She has taken opening medicines, with relief.

Fiat V.S. ad ℥ x:—Sumat Infus. Rosæ ℥ jss. c. Magnes. Sulphat. ʒ ij. ter die.—Applicetur Lotio Spirituosa (¼ spirit,) partibus affectis.—Diæta sit lactea.

April 14th.—The blood drawn on the 25th was very slightly inflamed, the coagulum being mottled on its surface. The spots faded on the 27th, and again returned on the 4th of April, with blood in the urine and stools. She was then purged with calomel, followed by sulphate of magnesia; and next day, the pulse having become full, though soft, and the abdomen tense, she was bled to the extent of eight ounces, in addition to the purging, which was repeated. After the venesection, the pulse became small and very frequent, and she continued very weak; a few spots appearing and disappearing from time to time. To-day, however, she complains of sickness, and severe pain and tenderness and tension across the umbilical portion of the abdomen. Motions very green; pulse 110, sharp; tongue furred, but moist; skin cool, very pale.

Fiat V.S. ad ℥ x.—H. Sal. effervescens c. Liq. Opii sedativ. m. iv. quartis horis.

15th.—Blood highly inflamed. Pain relieved by venesection, but the right iliac region is still very tender and tense. Pulse 110, sharp; tongue cleaner; skin warm; bowels open; nausea.

Repet. VS. ad ℥ x.—Peractâ venæsectione sumat Liquoris Opii sedativi m. xxv. ex Haustû effervescente ; et postea Haust. efferves. c. Liq. Opii sed. m. v. et Tinct. Digitalis m. x. sextis horis.—Dieta sit lactea.

26th.—The blood taken on the 15th was not inflamed, and the pain and tenderness were relieved; but, as they returned next day, sixteen leeches were applied to the abdomen; and, on the 21st, twelve ounces of blood were taken from the arm, in consequence of the pulse rising to 110, and becoming full and sharp. The purgatives have been continued. No new spots have appeared since the last report, *and she is now free from complaint.*

May 15th.—She remained pretty comfortable until the 12th instant, occasionally showing a few spots of purpura on the legs, which were immediately removed by calomel, with cathartics. On that day, however, she was attacked with violent pain in the occiput and back of the neck, accompanied with sense of throbbing in the head, and aggravated by the slightest motion, and attended also with occasional delirium. The pulse was small, frequent, and soft; skin warm and dry, but the countenance was pale and leucophlegmatic; the tongue slightly furred, but quite moist; and the bowels torpid.

She was twice cupped, and had leeches applied twice to the head. She had a bladder of ice constantly placed on the head, and blisters to the nape of the neck.—Purgatives, with Calomel, were freely administered, together with Digitalis, Tartarised Antimony, and Opium, with very little advantage.

—She became comatose this morning, her pupils being dilated; and died this afternoon.

*Sectio cadaveris, (next day.)—Abdomen*: Some of the convolutions of the small intestines conglutinated by perfectly formed adhesions, (evidently the produce of the inflammation of the peritoneum, which had been relieved by the treatment recorded in the earlier part of the history of the case.) No other abdominal disease was discovered.

*Thorax:* About an ounce of serum in the bag of the pericardium. The left ventricle of the heart dilated to nearly twice its natural size. The muscular parietes attenuated; one or two of the carneæ columnæ, however, seemed thickened. In the interior of the left auricle was observed a growth of a condylomatous character, of three-fourths of an inch in diameter. The mitral valve was much thicker than natural, but was not ossified.

*Head:* The whole arachnoid membrane, on the upper and back part of both hemispheres of the cerebrum, was covered with a lamina of coagulated lymph, evidently the product of the inflammatory attack which destroyed life.

